# Stem cell collection after lenalidomide, bortezomib and dexamethasone plus elotuzumab or isatuximab in newly diagnosed multiple myeloma patients: a single centre experience from the GMMG-HD6 and -HD7 trials

**DOI:** 10.1186/s12885-023-11507-9

**Published:** 2023-11-21

**Authors:** Joseph Kauer, Emma P. Freundt, Anita Schmitt, Niels Weinhold, Elias K. Mai, Carsten Müller-Tidow, Hartmut Goldschmidt, Marc S. Raab, Katharina Kriegsmann, Sandra Sauer

**Affiliations:** 1 Department of Haematology, Oncology and Rheumatology, University Hospital Heidelberg, Im Neuenheimer Feld 410, 69120 Heidelberg, Germany; 2 Molecular Medicine Partnership Unit (MMPU), Heidelberg, Germany; 3 GMMG Study Group at University Hospital Heidelberg, Heidelberg, Germany; 4 National Centre for Tumour Diseases (NCT), University Hospital Heidelberg, Heidelberg, Germany; 5 Laborarztpraxis, Laborarztpraxis Rhein-Main MVZ GbR, Limbach Gruppe SE, Frankfurt Am Main, Germany

**Keywords:** Multiple myeloma, Stem cell mobilization, Lenalidomide, Elotuzumab, Isatuximab

## Abstract

**Background:**

While quadruplet induction therapies deepen responses in newly diagnosed multiple myeloma patients, their impact on peripheral blood stem cell (PBSC) collection remains incompletely understood. This analysis aims to evaluate the effects of prolonged lenalidomide induction and isatuximab- or elotuzumab-containing quadruplet induction therapies on PBSC mobilization and collection.

**Methods:**

A total of 179 transplant-eligible patients with newly diagnosed MM treated at a single academic center were included. The patients were evaluated based on PBSC mobilization and collection parameters, including overall collection results, CD34^+^ cell levels in peripheral blood, leukapheresis (LP) delays, overall number of LP sessions, and the rate of rescue mobilization with plerixafor. The patients underwent four different induction regimens: Lenalidomide, bortezomib, and dexamethasone (RVd, six 21-day cycles, *n* = 44), isatuximab-RVd (six 21-day cycles, n = 35), RVd (four 21-day cycles, *n* = 51), or elotuzumab-RVd (four 21-day cycles, *n* = 49).

**Results:**

The patients' characteristics were well balanced across the different groups. Collection failures, defined as the inability to collect three sufficient PBSC transplants, were rare (*n* = 3, 2%), with no occurrences in the isatuximab-RVd and elotuzumab-RVd groups. Intensified induction with six 21-day cycles of RVd did not negatively impact the overall number of collected PBSCs (9.7 × 10^6^/kg bw versus 10.5 × 10^6^/kg bw, *p* = 0.331) compared to four 21-day cycles of RVd. Plerixafor usage was more common after six cycles of RVd compared to four cycles (16% versus 8%). Addition of elotuzumab to RVd did not adversely affect overall PBSC collection (10.9 × 10^6^/kg bw versus 10.5 × 10^6^/kg bw, *p* = 0.915). Patients treated with isatuximab-RVd (six cycles) had lower numbers of collected stem cells compared to those receiving RVd (six cycles) induction (8.8 × 10^6^/kg bw versus 9.7 × 10^6^/kg bw, *p* = 0.801), without experiencing significant delays in LP or increased numbers of LP sessions in a multivariable logistic regression analysis. Plerixafor usage was more common after isatuximab plus RVd compared to RVd alone (34% versus 16%).

**Conclusions:**

This study demonstrates that stem cell collection is feasible after prolonged induction with isatuximab-RVd without collection failures and might be further explored as induction therapy.

**Trial registration:**

Patients were treated within the randomized phase III clinical trials GMMG-HD6 (NCT02495922, 24/06/2015) and GMMG-HD7 (NCT03617731, 24/07/2018). However, during stem cell mobilization and -collection, no study-specific therapeutic intervention was performed.

## Introduction

In transplant-eligible patients with newly diagnosed multiple myeloma (MM), induction therapy followed by peripheral blood stem cell (PBSC) collection with granulocyte-colony-stimulating factor (G-CSF), high-dose chemotherapy (HDCT) with melphalan and autologous blood stem cell transplantation (ABSCT) is a standard-of-care [1–3].

While a single treatment with HDCT/ABSCT prolongs overall survival (OS) [4, 5], tandem treatment might improve outcomes even further and is used in some countries (i.e., Germany) [6, 7]. Patients that achieve a remission of more than 18 months after upfront HDCT/ABSCT may also benefit from salvage HDCT/ABSCT [8–10]. Therefore, up to three HDCT/ABSCTs may be performed during the treatment course of a MM patient. Accordingly, at our institution, PBSC collection by leukapheresis (LP) is considered successful if three sufficient transplants containing at least ≥ 2.0 × 10^6^ CD34^+^ cells/kg body weight (bw) have been collected [11, 12]. PBSC mobilization should be performed after induction therapy to ensure collection of a sufficient number of cells.

A variety of factors, such as higher age, melphalan-containing induction or previous radiotherapy involving haematopoietic bone marrow are associated with impaired PBSC collection results or increased rates of collection failure [13–15]. In contrast, the impact of lenalidomide induction on stem cell yield is a matter of debate [16–21].

Anti-CD38 monoclonal antibodies (mAb), such as daratumumab and isatuximab, significantly improve efficacy and outcomes after induction therapy [22–24]. However, various studies demonstrated a negative impact of daratumumab on PBSC collection [25–29]. The GMMG-HD7 multicentre study showed impaired overall stem cell collection after Isatuximab-RVd versus RVd (7.71 versus 9.54 × 10^6^/kg CD34^+^) without further detailed analyses [24]. Herein we report in-depth data on the effect of the anti-CD38 mAb isatuximab on PBSC collection. We further assessed the impact of intensified induction therapy with lenalidomide, bortezomib and dexamethasone (RVd, six 21-day versus four 21-day cycles) and the addition of the anti-SLAMF7 mAb elotuzumab to RVd on PBSC mobilization and collection parameters in patients treated within the randomized phase III clinical trials GMMG-HD6 (NCT02495922, 24/06/2015) and GMMG-HD7 (NCT03617731, 24/07/2018) [24, 30].

## Methods

### Patient selection and data collection

MM patients that were subjected to autologous PBSC collection at the Department of Haematology, Oncology and Rheumatology at the University Hospital Heidelberg within the clinical trials GMMG-HD6 and GMMG-HD7 between 2015 and 2021 were included (*n* = 179; HD6 = 100 patients, HD7 = 79 Patients). Patients underwent PSBC collection after mobilization chemotherapy with cyclophosphamide, doxorubicin, and dexamethasone (CAD) or cyclophosphamide. Details for each regimen are given in Table 1.

**Table 1:** Induction and mobilization therapy

Induction protocol	Dose	Application	Treatment days
**(isatuximab)-RVd (21 days/cycle, 6 cycles)**			
(Isatuximab)	10 mg/kg	iv	cycle 1: 1, 8, 15 cycle 2: 1, 8 cycle 3, 5: 1, 15 cycle 4, 6: 1
Lenalidomide	25 mg	po	1–14
Bortezomib	1.3 mg/qm	sc	1, 4, 8, 11
Dexamethasone	20 mg	po	cycle 1,3,5: 1, 2, 4, 5, 8, 9, 11, 12, 15 cycle 2,4,6: 1, 2, 4, 5, 8, 9, 11, 12
**(elotuzumab)-RVd (21 days/cycle, 4 cycles)**			
(Elotuzumab)	10 mg/kg	iv	cycle 1: 1, 8, 15 cycle 3–4: 1, 11
Lenalidomide	25 mg	po	1–14
Bortezomib	1.3 mg/qm	sc	1, 4, 8, 11
Dexamethasone	20 mg	po	1, 2, 4, 5, 8, 9, 11, 12, 15,
**Mobilization protocol**			
**CAD (28 days/cycle, 1 cycle)**			
Cyclophosphamide	1000 mg/qm	iv	1
Doxorubicin	15 mg/qm	Iv	1–4
G-CSF	10 µg/kg bw	iv	9,10,11,12,13,14
**Cyclophosphamide mono (28 days/cycle, 1 cycle)**			
Cyclophosphamide	1000 mg/qm	iv	1,2
G-CSF	10 µg/kg bw	iv	9,10,11,12,13,14

*G-CSF* Granulocyte colony-stimulating factor, *iv* Intravenous, *po* Per os, *qm* Square meter, *sc* Subcutaneous, *RVd* Lenalidomide, bortezomib, dexamethasone

Patients characteristics at first diagnosis, first line treatment, remission status, and detailed assessment of PBSC mobilization and collection results were collected retrospectively from routine medical records. Patient characteristics from the GMMG-HD7 trial were collected from study records.

### PBSC mobilization and collection

PBSC mobilization and collection by LP was performed according to protocols as previously described [31]. Mobilization protocols are shown in Table 1. Collection of three transplants comprised of ≥ 2.0 × 10^6^ CD34^+^ cells/kg bw was defined as successful collection. G-CSF (10 µg/kg bw) was applied on days 9—14. On day 14, the first PB CD34^+^ cell measurement was conducted. LP was initiated if the PB CD34^+^ cell count exceeded 10/µl. In the absence of infection or other limiting factors, the following LPs were conducted until collection of three transplants comprised of ≥ 2.0 × 10^6^ CD34^+^ cells/kg bw. In case of collection failure, reflected by insufficient PB CD34^+^ cell counts or insufficient collection, plerixafor was applied. In short, PB CD34^+^  < 10/µl after continued G-CSF stimulation until the day after the first planned measurement triggered pre-emptive plerixafor application. At PB CD34^+^ 10/µl—20/µl, plerixafor was used per treating physician’s discretion. Rescue mobilization was applied if less than 2.0 × 10^6^ CD34^+^ cells/kg bw were collected during LP1. Key metrics for evaluation of PBSC mobilization and collection include CD34^+^ cell counts/µl in the peripheral blood, collection delays due to poor mobilization, increased number of LP sessions due to insufficient collection results, collection of CD34^+^ cells/ kg bw upon the first session and CD34^+^ cell collection result upon all sessions.

### Procedures and definitions

Patients aged ≥ 18 years with untreated multiple myeloma requiring systemic therapy according to International Myeloma Working Group (IMWG) criteria [32] were included in the above mentioned trials. Detailed eligibility criteria are listed in the manuscript by Goldschmidt et al. reporting the primary end point of the GMMG-HD7 trial [24]. Response assessment was conducted according to IMWG criteria with near complete response as additional criterion [33]. Cytogenetic abnormalities were classified as high-risk in case of del(17)(p13), t(4;14)(p16;q32), or t(14;16)(q32;q23) in ≥ 10% of cells.

### Statistical analysis

Descriptive statistics were performed by R-Studio (R version 4.0.0, 2020–04-24) and SPSS (SPSS version 27). Data are depicted as absolute numbers and percentages, medians and ranges or means and standard deviations (SD). Categorical variables were compared using the Chi-Square test. Group means of continuous variables were compared by an analysis of variance (ANOVA). Median values of not normally distributed variables were compared by Kruskal–Wallis tests. Multivariable logistic regression analysis was performed with SPSS using the following dependent variables: Overall CD34^+^ collection results (≥ 10 × 10^6^/kg bw versus < 10), CD34^+^ cells in PB ((≥ 50/µl versus < 50/µl), LP delay (≥ 1 day versus 0 days), LP sessions (≥ 2 versus 1). The following independent variables were included: Age (> 60 versus ≤ 60 years), High-risk cytogenetic (yes versus no), ISS (3 versus 1–2), Induction six cycles of RVd vs. other, Induction Isa-RVd vs. other, Induction Elo-RVd vs. other and remission prior to mobilization.

(≥ VGPR versus < VGPR). *P* values < 0.05 were considered statistically significant.

## Results

### Patients’ characteristics and first line treatment

In this study, 179 patients that underwent induction therapy for newly diagnosed MM with subsequent PBSC mobilization and collection at our institution were included. Patients were grouped according to induction therapy: RVd (six 21-day cycles, *n* = 44), isatuximab-RVd (six 21-day cycles, *n* = 35), RVd (four 21-day cycles, *n* = 51), or elotuzumab-RVd (four 21-day cycles, *n* = 49). We aimed to assess the effect of intensified induction (six versus four cycles RVd), the addition of isatuximab to RVd (6 cycles) and the addition of elotuzumab to RVd (4 cycles) on PBSC mobilization and collection.

Patients characteristics at first diagnosis were equally distributed among groups, including gender, age, heavy chain type, light chain type, ISS stage, Salmon and Durie stage, and cytogenetic risk profile (Table 2). Response to induction therapy differed significantly between groups in favour of isatuximab-RVd (*p* = 0.006, Table 3).

**Table 2:** Patients` characteristics at first diagnosis

Variable	Overall cohort		RVd (six 21-day cycles)		Isatuximab –RVd (six 21-day cycles)		RVd (four 21-day cycles)		elotuzumab-RVd (four 21-day cycles)		*p* value
	**n**	**%**	**n**	**%**	**n**	**%**	**n**	**%**	**n**	**%**	
**Patient number**	179	100	44	100	35	100	51	100	49	100	/
**Gender**											0.808
Male	114	62	30	68	23	66	30	59	31	63	
Female	65	38	14	32	12	34	21	41	18	37	
**FIRST DIAGNOSIS**											/
**Diagnosis**											
MM	178	99	44	100	35	100	51	100	48	98	
Plasma cell leukaemia	1	1	0	0	0	0	0	0	1	2	
**Mean age at diagnosis, years (SD)**	58	8	58	9	58	7	56	9	58	7	0.615
**Heavy chain type**											0.523^a^
IgG	116	65	25	57	25	71	32	63	34	69	
IgA	32	18	11	25	5	14	6	12	10	20	
IgM	3	2	1	2	1	3	1	2	0	0	
IgD	0	0	0	0	0	0	0	0	0	0	
Double gammopathy	2	1	1	2	0	0	1	2	0	0	
Light chain only	26	15	6	14	4	11	11	22	5	10	
Non-secretory	0	0	0	0	0	0	0	0	0	0	
**Light chain type**											0.117^b^
Lambda	64	36	11	25	18	51	15	29	20	41	
Kappa	114	64	32	73	17	49	36	71	29	59	
Double gammopathy	1	1	1	2	0	0	0	0	0	0	
Non-secretory	0	0	0	0	0	0	0	0	0	0	
**ISS stage**											0.139
I	97	54	25	57	14	40	28	55	30	61	
II	41	23	7	16	14	40	11	22	9	18	
III	36	20	12	27	7	20	9	18	8	16	
NA	5	3	0	0	0	0	3	6	2	4	
**Cytogenetic profile**											0.793
High-risk	51	28	11	25	9	26	17	33	14	29	
Standard risk	114	64	29	66	24	69	30	59	31	63	
NA	14	8	4	9	2	6	4	8	4	8	

*Ig* Immunoglobulin, *ISS* International Staging System, *MM* Multiple myeloma, *NA* Not available, *PBSC* Peripheral blood stem cells, *SD* Standard deviation

^a^IgG versus IgA,IgD,IgM, Double Gammopathy versus Light chain only

^b^Lambda versus Kappa

**Table 3:** First line treatment

Variable	Overall cohort		RVd (six 21-day cycles)		isatuximab-RVd (six 21-day cycles)		RVd (four 21-day cycles)		elotuzumab-RVd (four 21-day cycles)		*p* value
	**n**	**%**	**n**	**%**	**n**	**%**	**n**	**%**	**n**	**%**	
**Patient number**	179	100	44	100	35	100	51	100	49	100	/
**Remission post induction**											**0.006**^a^
CR and nCR	52	29	22	50	12	34	10	20	8	16	
VGPR	76	42	10	23	16	46	25	49	25	51	
PR or worse	51	29	12	27	7	20	16	32	16	33	
**Median number of induction cycles (range)**	4	(4–6)	6	(4–6)	6	(4–6)	4	(3–4)	4	(3–4)	/

^a^CR + nCR versus VGPR versus PR or worse

*ABSCT* Autologous blood stem cell transplantation, *CR* Complete response, *HDCT* High-dose chemotherapy, *MR* Minimal response, *NA* Not available, *n.a.* Not applicable, *nCR* Near complete response, *PBSC* Peripheral blood stem cells, *PD* Progressive disease, *PR* Partial response, *RVd* Lenalidomide, bortezomib, dexamethasone, *SD* Stable disease, *VGPR* Very good partial response, *VCD* Bortezomib, cyclophosphamide, dexamethasone

### PBMC mobilization and collection

For the overall cohort the following PBSC mobilization and collection metrics were observed: either CAD (*n* = 167) or cyclophosphamide (*n* = 12) followed by G-CSF was applied for chemotherapy mobilization. Plerixafor application was performed, in general due to a delayed mobilization, in 25 patients (Table 4). Leukapheresis collection was considered successful if three transplants with a sufficient number of CD34^+^ cells (> 2 × 10^6^/ kg body weight) were collected. Collection failure occurred in three patients, with two of them being treated with six cycles RVd and one with 4 cycles RVd. Main outcome variables for PBSC collection were CD34^+^ cells in peripheral blood at first collection day, number of LP sessions, the need for plerixafor, LP delay due to poor mobilization as well as CD34^+^ collection results in the first session and all sessions.

**Table 4:** PBSC mobilization and collection

Variable	Overall cohort		RVd (six 21-day cycles)		isatuximab-RVd (six 21-day cycles)		RVd (four 21-day cycles)		elotuzumab-RVd (four 21-day cycles)		*p* value
	**n**	**%**	**n**	**%**	**n**	**%**	**n**	**%**	**n**	**%**	
**Patient number**	179	100	44	100	35	100	51	100	49	100	/
**Mobilization regimen**											/
CAD	167	93	39	89	28	80	51	100	49	100	
Cyclophosphamide	12	7	5	11	7	20	0	0	0	0	
**G-CSF dosage**											** < 0.001**
5 µg/kg bw/day	95	53	1	2	0	0	46	90	48	98	
10 µg/kg bw/day	84	47	43	98	35	100	5	10	1	2	
**Plerixafor application**											** < 0.001**
Yes	25	14	7	16	12	34	4	8	2	4	
No	154	86	37	84	23	66	47	92	47	96	
**Prolonged mobilization**											
Median delay, days (range)	0	(0–21)	0	(0–5)	0	(0–5)	0	(0–3)	0	(0–21)	0.785
Patients with distinct number of days in delay											0.777^a^
0	135	75	31	70	26	74	39	76	39	80	
1	28	16	9	20	5	14	9	18	5	10	
≥ 2	16	9	4	9	4	11	3	6	5	10	
**Blood count prior to first LP**											
Mean leukocyte count /nl (SD)	20	(12)	21	15	27	14	17	(8)	16	(8)	** < 0.001**
Mean PB CD34^+^ cells/µl (SD)	103	(75)	116	80	80	60	111	(91)	97	(57)	0.143
**First LP session**											
Mean CD34^+^ cells × 10^6^/kg (SD)	8,2	(4.2)	7,6	3,6	5,8	2,8	8,8	(4,8)	9,8	(4,1)	**0.004**
Mean processed blood volume, l (SD)	15,7	(3.8)	15,0	3,8	16,4	3,4	15,4	(4,2)	16,2	(3,3)	0.436
**Overall PBSC collection result**											
Mean CD34^+^ cells × 10^6^/kg	10,1	(2.9)	9,7	2,6	8,8	1,8	10,5	(3,5)	10,9	(3,0)	** < 0.001**
Collection failure, n (%)	3	2	2	5	0	0	1	2	0	0	0.301
**LP sessions**											
Median, n (range)	1	(1–3)	1	(1–3)	2	(1–3)	1	(1–3)	1	(1–3)	**0.008**
Patients with distinct number of LP sessions											**0.017**^b^
1	110	61	25	57	14	40	34	67	37	76	
2	59	33	18	41	17	49	14	27	10	20	
3	10	6	1	2	4	11	3	6	2	4	

*bw* Body weight, *CAD* Cyclophosphamide, doxorubicin, dexamethasone, *CR* Complete response, *G-CSF* Granulocyte-colony stimulating factor, *LP* Leukapheresis, *MR* Minimal response, *NA* Not available, *PBSC* Peripheral blood stem cells, *PD* Progressive disease, *PR* Partial response, *RVd* Lenalidomide, bortezomib, dexamethasone, *SD* Stable disease, *VCD* Bortezomib, cyclophosphamide, dexamethasone, *VGPR* Very good partial response

^a^0 days versus 1 day versus 2 or more days

^b^1 LP versus 2 LPs versus ≥ 3 LPs

### Effect of intensified induction on PBSC mobilization/collection

**Fig. 1:**
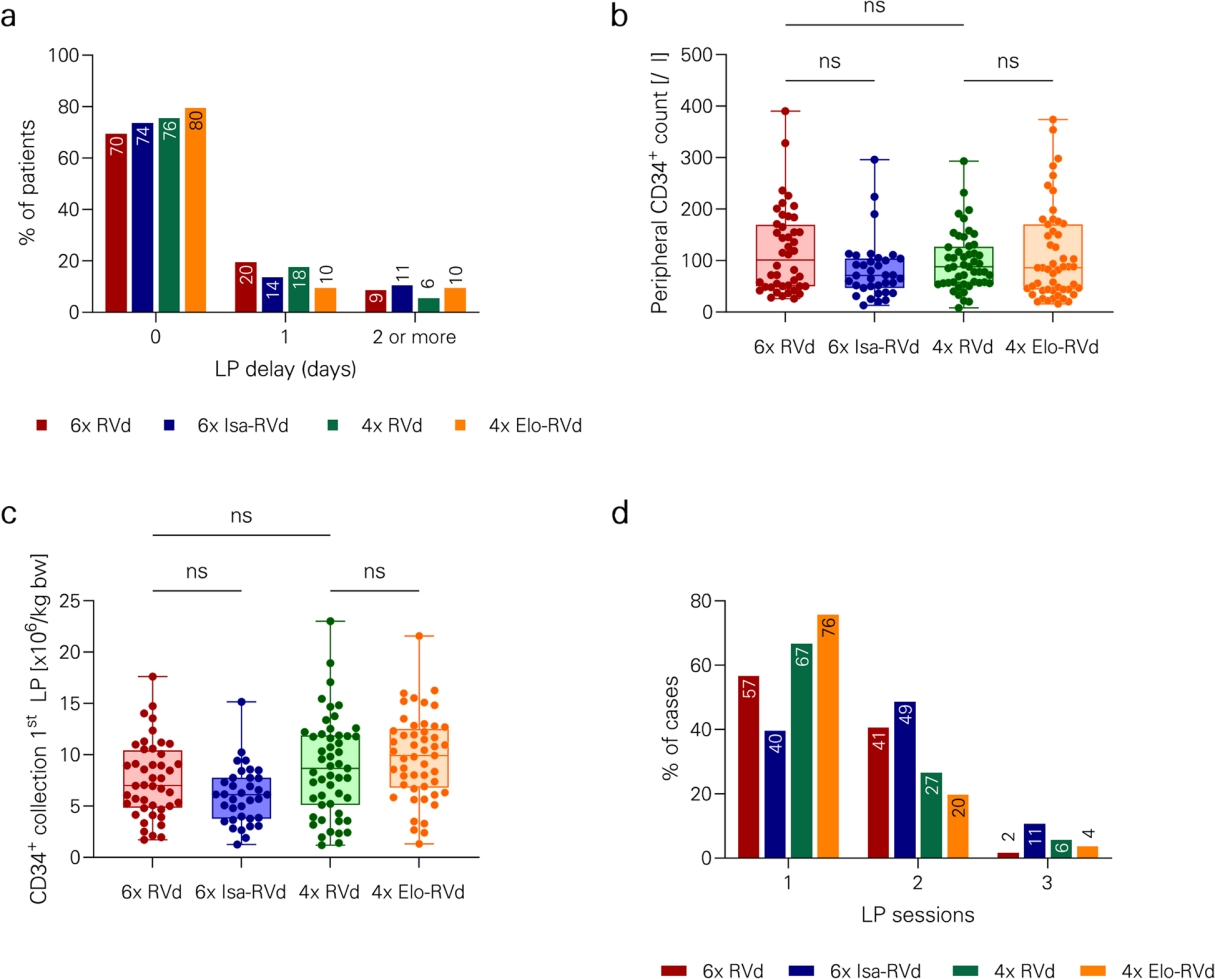
PBSC mobilization metrics. **a** Proportions of patients with delayed mobilization are shown. **b** Peripheral blood CD34^+^ cell counts at the first LP are depicted. **c** CD34 + cell collection results after the first LP session are shown. **d** Percentages of patients with distinct numbers of LP sessions are displayed. Abbreviations: bw, body weight; d, days; Elo, elotuzumab; Isa, isatuximab; LP, leukapheresis; PB, peripheral blood; PBSC, peripheral blood stem cells, RVd, lenalidomide, bortezomib, dexamethasone

**Fig. 2:**
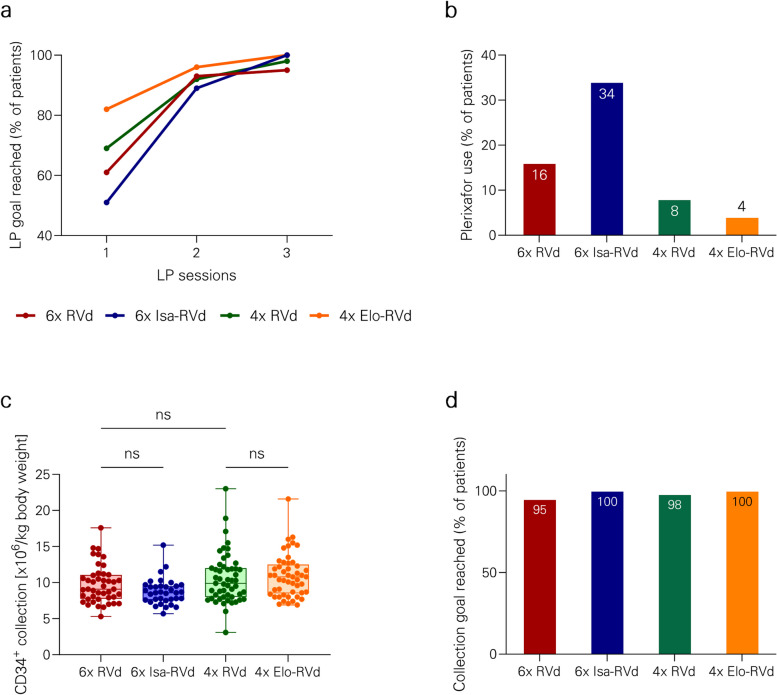
Overall PBSC collection results. **a** The percentages of patients reaching collection goal of > 6 × 10^6^ CD34 + cells /kg bw according to collection days are displayed. **b** The proportion of patients receiving pre-emptive or rescue mobilization with plerixafor is shown. **c** The overall CD34^+^ cell collection result after all LP sessions are shown. **d** Percentage of patients reaching the collection goal of > 6 × 10^6^ CD34 + cells /kg bw after all LP sessions are depicted. Abbreviations: bw, body weight; d, days; Elo, elotuzumab; Isa, isatuximab; LP, leukapheresis; PB, peripheral blood; PBSC, peripheral blood stem cells, RVd, lenalidomide, bortezomib, dexamethasone

First, we focused on the effect of intensified induction with prolonged RVd (six 21-day cycles) versus standard RVd (four 21-day cycles). Mean PB CD34^+^ cell count (116/µl versus 111/µl, *p* = 0.999), number of LP sessions (median 1 (range 1–3) versus 1 (range 1–3), plerixafor use (16% versus 8%), LP delay (median 0 (range 0–5) versus 0 days (range 0–3), *p* = 0.999), mean CD34^+^ cell collection result upon first LP (7.6 × 10^6^/kg bw, interquartile range [IQR] 5.1) versus 8.8 × 10^6^/kg bw, IQR 6.7, *p* = 0.335) and overall CD34^+^ cell collection result (9.7 × 10^6^/kg bw, IQR 3.1, versus 10.5 × 10^6^/kg bw, IQR 3.9, *p* = 0.331) did not significantly differ between these groups (Figs. 1, 2 and Table 4).

### Impact of quadruplet therapy on PBSC mobilization/collection

Next, the impact of addition of isatuximab to RVd (six 21-day cycles) was evaluated. Mean PB CD34^+^ cell count (80/µl versus 116/µl, *p* = 0.424), number of LP sessions (median 2 (range 1–3) versus 1 (range 1–3), *p* = 0.401), plerixafor use (34% versus 16%, *p* = 0.176), LP delay (median 0 (range 0–5) versus 0 days (range 0–5, *p* = 0.999), mean CD34^+^ cell collection result upon first LP (5.8 × 10^6^/kg bw, IWR 3.7 versus 7.6 × 10^6^/kg bw, IQR 5.1, *p* = 0.460) and overall CD34^+^ cell collection result (8.8 × 10^6^/kg bw, IQR 1.8 versus 9.7 × 10^6^/kg bw, IQR 3.1, *p* = 0.801) did not significantly differ between groups (Figs. 1, 2 and Table 4).

Addition of elotuzumab to RVd (four 21-day cycles) did not hamper PBSC collection results. Mean PB CD34^+^ cell count (97/µl versus 111/µl, *p* = 0.807), number of LP sessions (median 1 (range 1–3) versus 1 (range 1–3)), plerixafor use (4% versus 8%), LP delay (median 0 days (range 0–21) versus 0 days (range 0–3)), mean CD34^+^ cell collection result upon first LP (9.8 × 10^6^/kg bw, IQR 5.4 versus 8.8 × 10^6^/kg bw, IQR 6.7, *p* = 0.625) and overall CD34^+^ cell collection result (10.9 × 10^6^/kg bw, IQR 3.8 versus 10.5 × 10^6^/kg bw, IQR 3.9, *p* = 0.915) did not significantly differ between groups (Figs. 1, 2 and Table 4).

### Multivariate analysis

Multivariable logistic regression analysis was performed regarding the outcome variables mobilization delay, number of LP sessions, peripheral blood CD34^+^ levels and overall CD34^+^ cell collection results. Age > 60, High-risk cytogenetics, ISS stage 3 (versus 1–2), induction regimen and remission after induction (≥ VGPR versus < VGPR) had no significant impact on LP delay, LP sessions and peripheral blood CD34^+^ levels (Table 5). Induction with isatuximab-RVd significantly reduced the rate of exceptionally high overall collection results (≥ 10^7^/kg bw, Odds ratio 0.17, 95% CI 0.05–0.51, *p* = 0.002). However, no relative collection failure, defined by the inability to collect three sufficient PBSC transplants, was observed with isatuximab-RVd.

**Table 5:** Multivariable logistic regression analysis

**Variable**	**Overall CD34**^**+**^** collection results (≥ 10 × 10**^**6**^**/kg bw versus < 10)**		**CD34**^**+**^** cells in PB ((≥ 50/µl versus < 50/µl)**	
	**Odds ratio** **[95% CI]**	***p* value**	**Odds ratio** **[95% CI]**	***p* value**
**Age (> 60 versus ≤ 60 years)**	0.89 [0.45 -1.76]	0.743	0.68 [0.34 -1.38]	0.289
**High-risk cytogenetic (yes versus no)**	0.95 [0,41 -2.20]	0.904	1.14 [0.47 -2.80]	0.773
**ISS (3 versus 1–2)**	1.50 [0.72 -3.09]	0.279	1.07 [0.50 -2.30]	0.854
**Induction 6 × RVd vs. other**	0.88 [0.37 -2.09]	0.770	1.22 [0.49 -3.03]	0.671
**Induction 6 × Isa-RVd vs. other**	0.17 [0.05 -0.51]	**0.002**	1.53 [0.57 -4.07]	0.399
**Induction 4 × Elo-RVd vs. other**	1.24 [0.54 -2.88]	0.611	2.59 [0.97 -6.89]	0.057
**Remission prior to mobilization** **(≥ VGPR versus < VGPR)**	1.39 [0.66 -2.90]	0.384	1.34 [0.62 -2.90]	0.460
**Variable**	**LP delay (≥ 1 day versus 0 days)**		**LP sessions ((≥ 2 versus 1)**	
	**Odds ratio** **[95% CI]**	***p* value**	**Odds ratio** **[95% CI]**	***p* value**
**Age (> 60 versus ≤ 60 years)**	1.71 [0.79 – 3.70]	0.172	1.03 [0.52 -2.04]	0.929
**High-risk cytogenetic (yes versus no)**	2.37 [0.98 -5.73]	0.055	0.80 [0.34 -1.87]	0.594
**ISS (3 versus 1–2)**	1.20 [0.51 -2.80]	0.677	1.55 [0.74 -3.26]	0.247
**Induction 6 × RVd vs. other**	1.72 [0.62 -4.72]	0.296	1.69 [0.69 -4.12]	0.252
**Induction 6 × Isa-RVd vs. other**	1.30 [0.43 -3.92]	0.646	2.47 [0.97 -6.30]	0.058
**Induction 4 × Elo-RVd vs. other**	0.80 [0.27 -2.41]	0.693	0.61 [0.24 -1.57]	0.305
**Remission prior to mobilization** **(≥ VGPR versus < VGPR)**	0.76 [0.32 -1.78]	0.528	0.79 [0.38 -1.66]	0.531

*CI* Confidence interval, *Elo* Elotuzumab, *Isa* Isatuximab, *ISS* International Staging System, *LP* Leukapheresis, *PB* Peripheral blood, *RVd* Lenalidomide, bortezomib, dexamethasone, *VGPR* Very good

## Discussion

This academic single centre study provides novel data on the impact of different state-of-the-art induction regimen on PBSC mobilization and collection metrics in patients with newly diagnosed MM. While several factors have been described as being harmful to PBSC collection [13–15], no such data are available for quadruplet induction therapies comprising isatuximab and comparisons of different lengths of RVd induction. This study was able to assess important direct and indirect parameters of successful stem cell collection such as LP delay, number of LP sessions, plerixafor utilization, and overall collection results.

Quadruplet induction therapies such as daratumumab, lenalidomide, bortezomib, and dexamethasone (Dara-RVd) or daratumumab, bortezomib, thalidomide, and dexamethasone (Dara-VTd) are now standard-of-care for transplant-eligible patients with newly diagnosed multiple myeloma [22, 23]. Since CD38 is expressed on CD34^+^ progenitor cells [34], concerns regarding impaired stem cell mobilization after CD38-targeting antibody therapy have been raised. A negative impact of daratumumab on PBSC mobilization and collection has been described in the setting of several clinical trials. Within the phase III CASSIOPEIA trial, overall stem cell collection was impaired after Dara-VTd compared to VTd (6.7 vs. 10.0 × 10^6^/kg bw), additionally mirrored by increased utilization of plerixafor (21.7 vs. 7.9%) and higher rates of relative collection failure (reported as collection < 5 × 10^6^/kg bw, 24.6% vs. 11.4%) [35]. Though our clinical practice is similar and includes cyclophosphamide-based mobilization chemotherapy and a rescue policy including plerixafor, the collection failure rate in the CASSIOPEIA trial was higher compared to our study.

In the phase II GRIFFIN trial, lower stem cell yield (8.3 versus 9.4 × 10^6^/kg bw) and higher utilization of plerixafor (72% vs. 55%) was seen after daratumumab plus RVd versus RVd alone [26]. However, institutional practice regarding plerixafor rescue or upfront application differed between participating centres, with some using steady-state mobilisation. Furthermore, cyclophosphamide mobilization chemotherapy was only permitted after unsuccessful mobilization with G-CSF with or without plerixafor. In patients that underwent a rescue plerixafor strategy similar to the strategy employed at our centre, 41% of patients received plerixafor after daratumumab-RVd versus 27% after RVd [26].

In contrast to the data on daratumumab, our results suggest that isatuximab does not increase the risk for relative collection failure although the total number of collected stem cells is lowered. Furthermore, utilization of plerixafor was required in a minority of patients (34%) and upfront application to all patients might not be necessary after 18 weeks of isatuximab-RVd, thus limiting the economic burden of this regimen. However, a direct comparison between daratumumab and isatuximab regarding stem cell collection yield cannot be drawn from our data. The observation is in line with an extensive in vitro study, in which isatuximab did not induce bone marrow toxicity in vitro while effectively lysing MM cells [36]. The multicentre data of the GMMG-HD7 trial showed a significantly impaired overall collection after induction therapy isatuximab-RVd versus RVd alone (7.71 versus 9.54 × 10^6^/kg CD34^+^). The smaller gap in overall collection rate at our centre after isatuximab-RVd versus RVd alone (8.8 versus 9.7 × 10^6^/kg CD34^+^) might be explained by the extensive experience and high patient volume. Some patient characteristics might have been beneficial for stem cell collection in our cohort, such as the relatively low median age (58 years) and the use of mobilization chemotherapy in all patients. Furthermore, collection was considered successful after collection of at least 6 × 10^6^/kg bw, which might be lower than collection goals in other studies.

This study also aimed to compare induction regimen in newly diagnosed MM patients receiving lenalidomide in either a standard regimen (4 cycles, 25 mg/day for 14 days) or a prolonged regimen (6 cycles, 25 mg/d for 14 days). No significant differences regarding PBSC mobilization and collection metrics were observed in our study. The previously reported data on lenalidomide treatment prior to PBSC collection reveals contradictory results. Initially, Kumar et al. suggested a negative impact of lenalidomide on stem cell collection in patients treated with lenalidomide-dexamethasone [16]. This was confirmed by Bhutani et al., who found that lenalidomide application over eight or more courses correlates with poor collection results and increased number of LP sessions [17]. More recent data on lenalidomide in a small cohort receiving also RVd suggests delayed mobilization and increased numbers of LP sessions [18]. Another recent study, in contrast, did not reveal any negative effects of prolonged lenalidomide exposure (> 6 cycles) on LP results [20]. Of note, the latter study comprised a cohort of patients treated with a variety of different induction regimens containing lenalidomide, which hampers direct comparisons. The standardized lenalidomide-containing induction therapies in our cohort ensure comparability and allow for multivariate analyses, thus reducing confounders.

The SLAMF7 antibody elotuzumab is an established therapeutic option in relapsed MM [37, 38]. While being present on MM cells, SLAMF7 is not expressed on other bone marrow cells [39]. Detailed data on the impact of elotuzumab treatment prior to PBSC transplantation are missing. We here provide evidence that elotuzumab does not affect PBSC mobilization and collection metrics, which is in line with the multicentre data from the GMMG-HD6 trial [40]. Likely, due to the negative results of the SWOG-1211 and the GMMG-HD6 trials, elotuzumab will not be utilized in the front-line setting combined with RVd in transplant-eligible patients with newly diagnosed MM [30, 41]. However, studies combining elotuzumab with other regimen in the front-line setting are ongoing.

Limitations of our study include its single centre design and its retrospective nature. While patients were treated within randomized trials, stem cell collection was not an endpoint of either trial. Comparisons with outcomes in other trials or other centres might therefore be impaired. The collection results presented represent outcomes after quadruplet therapy followed by cyclophosphamide-based mobilization chemotherapy and might not be transferable to steady-state mobilization.

## Conclusions

In summary, our study demonstrated that stem cell collection is feasible after prolonged induction with isatuximab-RVd and did not lead to collection failure in this academic single centre cohort. Moreover, induction therapy with RVd (21-days) for four or six cycles did not negatively impact overall collection results.

## Author information

Not applicable.

## Data Availability

All data generated or analysed during this study are included in this published article. The GMMG-HD7 trial is ongoing. Data from published parts of the trial can be made available upon reasonable request to the principal investigator (HG; hartmut.goldschmidt@med.uni-heidelberg.de) and the board of directors of the GMMG study group.
